# Influence of Self-Esteem of Middle School Students for Mental Care on Academic Achievement: Based on the Mediation Effect of GRIT and Academic Enthusiasm

**DOI:** 10.3390/ijerph18137025

**Published:** 2021-06-30

**Authors:** Jhong Yun (Joy) Kim, EunBee Kim, InSu Lee

**Affiliations:** 1 BK21FOUR Group of Education, Korea University, 145 Anam-Ro, Anam-Dong, Seongbuk-Gu, Seoul 02841, Korea; joycello@hanmail.net; 2 Department of Education, Korea University, 145 Anam-Ro, Anam-Dong, Seongbuk-Gu, Seoul 02841, Korea; smile9061@naver.com

**Keywords:** middle school students, mental care, self-esteem, academic achievement, GRIT, academic enthusiasm

## Abstract

The purpose of this study is to identify how self-esteem of middle school students for mental care influences their academic achievement and to verify the mediation effect of GRIT on academic enthusiasm. Data of 2590 first graders in middle school from the Kora Children and Youth Panel Survey 2019 was used to support this study. Data analysis was performed by using SPSS21.0, AMOS22.0, and PROCESS macro program. The results are as follows. Comparison of the model fits of each full mediation model and partial mediation model with χ^2^ showed that the full mediation model was more suitable for this study. In more detail, the influence of self-esteem on GRIT and the influence of GRIT on academic enthusiasm were significantly positive. Lastly, the study identified that there was a mediation effect between self-esteem and academic achievement through GRIT and academic enthusiasm. It indicates that self-esteem is the key to improve academic achievement and that specific programs should be supplemented in order to enhance self-esteem, GRIT, and academic enthusiasm.

## 1. Introduction

Entrance exams heavily influence the education system in Korean society. This applies to middle school students who are evaluated primarily on their academic achievements [1]. Academic achievement not only determines their advancement to high school and college, but also serves as a strong foundation for them to succeed in society after graduation.

Middle school is a period when students experience great changes in their physical, emotional, and social development [2]. This is especially true for middle school students who must adjust to a different school environment during their adolescent years [3]. During this period, building self-esteem is important to both overcoming hardships and to establish a positive self-image [4]. Self-esteem is a primary measure of how middle school students are evaluated, viewed, and respected as a person [5]. Among previous studies conducted in regards to self-esteem and academic achievement, there is research indicating that self-esteem has high predictive power on Korean literature, English, mathematics, and science subjects [6], and one suggesting how more high school students recognize that self-esteem reinforces their attitude to learn different subjects [7].

According to previous studies, there were many cases where academic achievement meant cognitive area in the field of school [8]. Academic achievement includes grades and academic background of subjects granted to individual students, and there are results indicating that students attending prestigious middle schools achieved outstanding academic achievements [9]. However, such academic achievement is closely related to the mental health of individuals, and accumulated failure in academic learning tends to be a reason for negative self-esteem [10]. According to studies by Hwang et al. [11], middle school students with high academic achievements experienced greater psychological hardship due to academic learning. Gu [12] argues that academically outstanding students tend to have more anxiety or negative emotion versus regular students, which supports the correlation between self-esteem and psychological with academic achievement. Other studies also support this correlation [5,13].

As the correlation between definitive and cognitive characteristics has recently been defined in the field of educational study, there has been active research on definitive characteristics. GRIT is one of the representative variables of definitive characteristics. GRIT is defined as passion and effort that are maintained for a long time. The higher the level of GRIT, the greater tendency to continuously make an effort to achieve goals [14,15]. Previous research has shown that college students with high GRIT were reported to show outstanding academic achievements. For example, in regards to retention of cadets in the military academy or prediction of high-salaries, GRIT was reported to be a greater predictive power over intelligence. Lee [16] writes that GRIT is a great indicator of success and self-esteem. There is research that points to low self-esteem and emotional problematic behaviors [17,18].

This study aims to examine GRIT, which has recently emerged in importance as a variable that can enhance mental care in adolescents. GRIT means the power to continue to achieve long-term goals despite adversity and difficulties. GRIT is a non-cognitive personality trait consisting of persistence to goal and persistence of interest, consistency in effort is behavioral persistence, and a tendency to continue to work without giving up even if one encounters difficulties in moving toward long-term goals. On the other hand, persistence of interest corresponds to a motivational aspect passion) and is a tendency to consistently sustain it without losing interest toward the goal as we move forward to achieve it [19]. Furthermore, GRIT has been reported to play a positive role not only in the level of individual function but also in the promotion of individual well-being, including psychological health and satisfaction with life [20]. Specifically, people with higher GRIT experience less psychological exhaustion and depression [21,22], while experiencing more positive emotions [23].

Recent studies have shown that effort and persistence have a significant impact on academic achievement, unlike previous reports [12] that innate factors such as intelligence are important in academic achievement. Duckworth et al. [14] found common characteristics in individuals with similar innate intelligence and talent but different accomplishments than others, calling them “GRIT”, a long-term effort and determination to achieve goals set by individuals, especially for students’ mental care. Ericsson and Ward also found that people with high grits can work hard on the task, maintain mental health in the face of hardship, adversity, and failure, and that despite similar abilities in recent studies [12], students are more mentally fit and overcome depression. However, with regard to middle school students’ mental care, studies of the relation between their academic achievement and emotions and the correlation between mental health factors such as grit are insufficient.

On the other hand, academic enthusiasm is referred to as a high degree of energy and emotional resilience to learn [24]. According to Korean Children and Youth Panel Survey [25], while middle school students were devoted and focused on their studies, their academic enthusiasm turned out to be about 60% [2]. Previous studies conducted on academic enthusiasm points to a wide array of other variables to include the following: academic stress [26,27], perfectionism [28], interest in PE class [29], peer relationship [29,30], academic burnout [31], learning motivation, and roles of instructors and subjects [32]. Some non-subjective factors include sincerity, energy, amicable situations, and social support [33].

In summary, there is ample evidence that the more middle school students build self-esteem, the more likely it was for them to achieve academic success. GRIT and academic enthusiasm were known as key factors related to academic achievement. However, since there were no previous studies conducted on the role of academic enthusiasm on self-esteem and academic achievement, the results are inconclusive. According to the study conducted by Shin and Kang [2] on the influence of social withdrawal of middle school students on academic enthusiasm, self-esteem turned out to have an intermediary role.

Hereupon, this study aims to identify the influence of self-esteem of middle school students for mental care on academic achievements and verify the mediation effect of GRIT and academic enthusiasm in it. Research questions to explore this study are as follows.

Research question 1. How does self-esteem of middle school students influence GRIT, academic enthusiasm, and academic achievement?

Research question 2. Will GRIT and academic enthusiasm of middle school students affect self-esteem and academic achievement?

### 1.1. Theoretical Background

#### 1.1.1. Self-Esteem

Self-esteem is how a person evaluates him/herself to be a valuable individual. It is a self assessment. An individual with high self-esteem tends to be satisfied, and they view their strengths and weaknesses positively. However, a low level of self-esteem makes a person focus more on their weakness. Ryu [34] has viewed self-esteem to be related to emotional factors such as good or bad.

Coopersmith [35] emphasized the importance of self-esteem during the adolescent period. In this period, adolescents experience changes in educational environment as well as physical maturity, and hence in the development stage, to judge their values or positions [34]. According to research by Rosenberg [5], juveniles with low self-esteem tended to evaluate themselves as lacking talents required to succeed even if they had ambitious career goals. As a result, they tended to have noticeable anxiety, depression, low level of achievement, and social isolation. On the other hand, juveniles with high self-esteem showed positive views of their talents, leadership, intelligence, social skills, and ability to show good impression [36].

Yeon [37] wrote that juveniles with high self-esteem are able to view themselves with, love themselves, have firm belief, and are able to move forward. On the other hand, juveniles with low self-esteem tends to avoid new challenges due to anxiety and fear of failure.

There is an urgent need to build high self-esteem among juveniles. Since juveniles are able to overcome their situations based on beliefs in themselves, reinforcing it will increase a chance for success.

#### 1.1.2. GRIT

There have been many research studies conducted on the effect of personal characteristics or capabilities on academic achievement or performance. However, they have all focused on short-term performance. Duckworth et al. [19] mentioned GRIT as a predictive variable on long-term success. GRIT is defined as a continuous effort exerted to accomplish long-term goals and is relevant to two sub-categories: consistency of interest and perseverance of effect. In addition, consistency of interest is defined as the ability to maintain interest in a certain goal or a concern on clarifying the relationship among personal GRIT, subjective inference, and efficiency of self-control learning in the long-term perspective on a goal in corresponding to social expectations. Perseverance is related to coping with situations to achieve goals in a flexible manner while controlling behaviors of individuals [38]. GRIT seems to be similar to sincerity or self-control. However, while it focuses on short-term accomplishment, GRIT pursues long-term goals based on a self-led decision-making process.

In addition, the goal of GRIT is not clearly shown but is in the same context with mastering goals. Mastering goals is defined as goal-oriented to focus on improving abilities while learning new knowledge or skills. Therefore, it contributes to academic achievement and performance [39]. However, if mastering goals is regarded as an attitude related to achieving goals, GRIT is a key variable as it focuses on achieving goals while including definitive and behavioral characteristics. In addition, unlike the common belief that intelligence or ability is a determining factor for performance, long-maintained practice has been proven to be a greater indicator on performance than intelligence [40,41]. Therefore, GRIT is indeed an important variable to predict performance. According to previous studies, GRIT has shown to have a high predictive power on variables related to academic achievements of college students, retention of cadets at military academy, and performance/retention of teaching professionals.

Additionally, GRIT has also been shown to be associated with individuals’ mental health and well-being [24]. GRIT has been associated with depression, which is a negative indicator of mental health, and euphoria and life satisfaction [16]. In Duckworth et al. [19]’s work, fighting spirit was related to extroversion and static relationships, and to neuroticism. According to a study conducted on high school students by Datu et al. [21]. GRIT was associated with low levels of depression as a medium for meaning in life. GRIT is likely to lead to obtaining a positive experience from difficulties and failures, thereby reducing mental health.

#### 1.1.3. Academic Enthusiasm

Academic enthusiasm has been approached differently by scholars. Schaufeli et al. [24] defined academic enthusiasm to be constituted with vigor, dedication, and absorption in an opposite concept of academic burnout. Leiter and Harvie [42] classified enthusiasm as energy, involvement, and efficacy. In addition, González-Romá et al. [43] viewed it as an important factor on enthusiasm, vigor, and dedication. As shown above, there has been continuous controversy on components and concepts on enthusiasm, while raising the need for empirical study. In addition, those with high levels of academic enthusiasm turned out to use various cognitive strategies in their academic achievement [44,45].

Learners are motivated by academic enthusiasm when it comes to participation [46]. Therefore, academic enthusiasm is an important element to examine successful school life [47] and continued participation [48]. According to previous studies, academic enthusiasm has been defined in diverse terms such as academic flow or learning participation [48,49]. Among them is one by Mosher and MacGowan [50] who defined the concept of enthusiasm to be an attitude of leading learning, behaving, and participating. Kahn [51] defined it as a behavior to proactively participate in learning. This implies that enthusiasm is inherent in behavioral practice of learning. On the other hand, participation and flow are somewhat different in the behavioral perspective. Participation is a comprehensive concept that consists of multi-dimensional elements including motivation and flow, while flow is viewed as an optimal experience of individuals from enthusiasm and participation [52]. In summary, participation and flow encompass a more cognitive and emotional concept compared to enthusiasm.

#### 1.1.4. Academic Achievement and the Relationship with Variables

Academic achievement is known to be accomplished through continuous effort. Therefore, the relationship to GRIT has long been a concern. There are researches studies [53] indicating how academic achievement and GRIT are positively correlated, and also ones [54,55] showing how GRIT predicts academic achievement in high school students. In addition, one research study [56] conducted on academic self-control of elementary school students as deeply related to academic achievement, or the ones [56,57] on academic adaptation among adult learners, it is expected that students with high GRIT can accomplish a high level of academic achievement. However, recent studies have reported a low influence of GRIT on academic achievement. For example, GRIT suggests an effect of deliberate practice as a behavioral mechanism that influences achievement. Deliberate practice is a very difficult course as learners internalize external feedback and experience a process to observe, monitor, and control their performance [58]. According to the research by Lee [16], deliberate practice provides a connection between GRIT and academic achievement.

Self-esteem is an evaluation of one’s value. Rosenberg [5] defined self-esteem as either a positive or negative oneself assessment. In other words, how middle school students view themselves may influence not only academic achievement, but other behaviors. According to previous studies, self-esteem was reported to be a factor on academic achievement and ability to adjust at school [59,60]. According to a prior study, students with high self-esteem better adapted to school and had higher or academic achievement. However, students with low self-esteem had a high level of anxiety and abnormal behaviors. On the other hand, previous studies indicate self-esteem as a factor influencing academic achievement, as in the more a person positively views themselves during adolescence, the more likely it was for them to learn with attainable goals. According to a study by Son et al. [61], dealing with self-control and academic achievement among juveniles, positive self-evaluation was reported to be the factor on academic achievement.

Learners tend to continue their learning activity participation selectively depending on their level of academic enthusiasm [46]. Since academic enthusiasm is the main element in examining successful school life [47] and continuity in learning participation [48], the positive relationship between academic enthusiasm and academic achievement is expected.

## 2. Methods

### 2.1. Research Model

**Figure 1:**
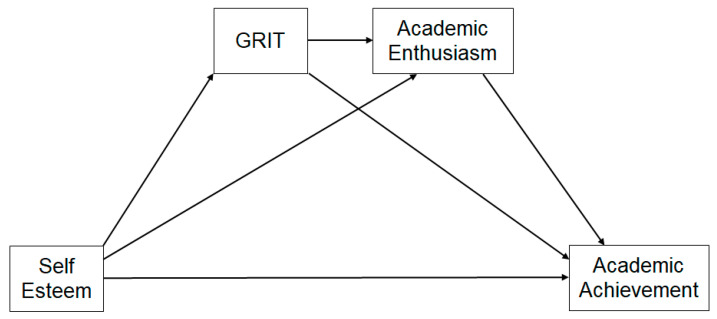
Research model.

This study aimed to see whether there was a mediation effect of GRIT and academic enthusiasm in the relationship between self-esteem and academic achievement among middle school students. This research is shown in Figure 1.

### 2.2. Data Collection and Participants

This study was conducted on 2590 middle school first graders using data in the Korea Children and Youth Panel Survey 2018 (KCYPS 2018) completed by the National Youth Policy Institute. KCYPS 2018 was designed to establish data to view complex changes regarding growth and development of children and youth in systemic and multi-dimensional perspective with support of Ministry of Education, Korea. This panel survey was reviewed with the National Youth Policy Institute IRB (Institutional Review Board) and executed using TAPI (Tablet Assisted Personal Interview) method for multi-stage stratified sampled middle school first graders.

Among the 2590 participants, there were 1409 (54.2%) male and 1185 female participants (45.8%). There were 1169 (45.1%) students attending middle school in metropolis, 1054 students (40.7%) attending middle school in small or medium-sized cities, and 367 students (14.2%) attending middle school in rural areas. A statistical breakdown on the regions included Gyeonggi (614 students, 23.7%), Seoul (426 students, 16.4%), and Incheon (197 students, 7.6%). In addition, the average age of the participants was 13 years old (M_age_ = 13.052).

### 2.3. Measures

Within this panel data, questionnaire responses on self-esteem, grit, academic enthusiasm, and the academic achievement of first-grade students in middle school were used for this study. The instruments of this study were obtained from previous studies. The scale range was from 1 (strongly disagree) to 4 (strongly agree). To examine the adequacy of the scales, inter-correlations were taken.

Self-esteem was measured using the Rosenberg’s [5] self-esteem scale, translated by National Youth Policy Institute. Participants were asked to estimate their self-esteem through items such as “I feel that I have a number of good qualities”, “I am able to do things as well as most other people”, and “I take a positive attitude toward myself”.

To measure GRIT, the Korean GRIT scale for children developed by Kim and Hwang [62] was used. This scale was composed of eight items and confirmed the reliability from 309 6th grade students in Korea. Sample items include “Once I start something, I unconditionally finish it” and “I am diligent”.

For academic enthusiasm measurement, Korean Academic Engagement Inventory (KAEI) developed by Lee and Lee [63] was used. This scale consisted of 16 items; four items for each four dimension of vigor, dedication, esteem, and immersion. The sample questionnaires to measure academic enthusiasm include “I’m good at studying”, “I am confident in my studies”, and “I became energetic while I’m studying”.

The academic achievement questionnaires consisted of two items—The subjective academic achievement level and academic achievement satisfaction level—developed by the National Youth Policy Institute.

**Figure 2:**
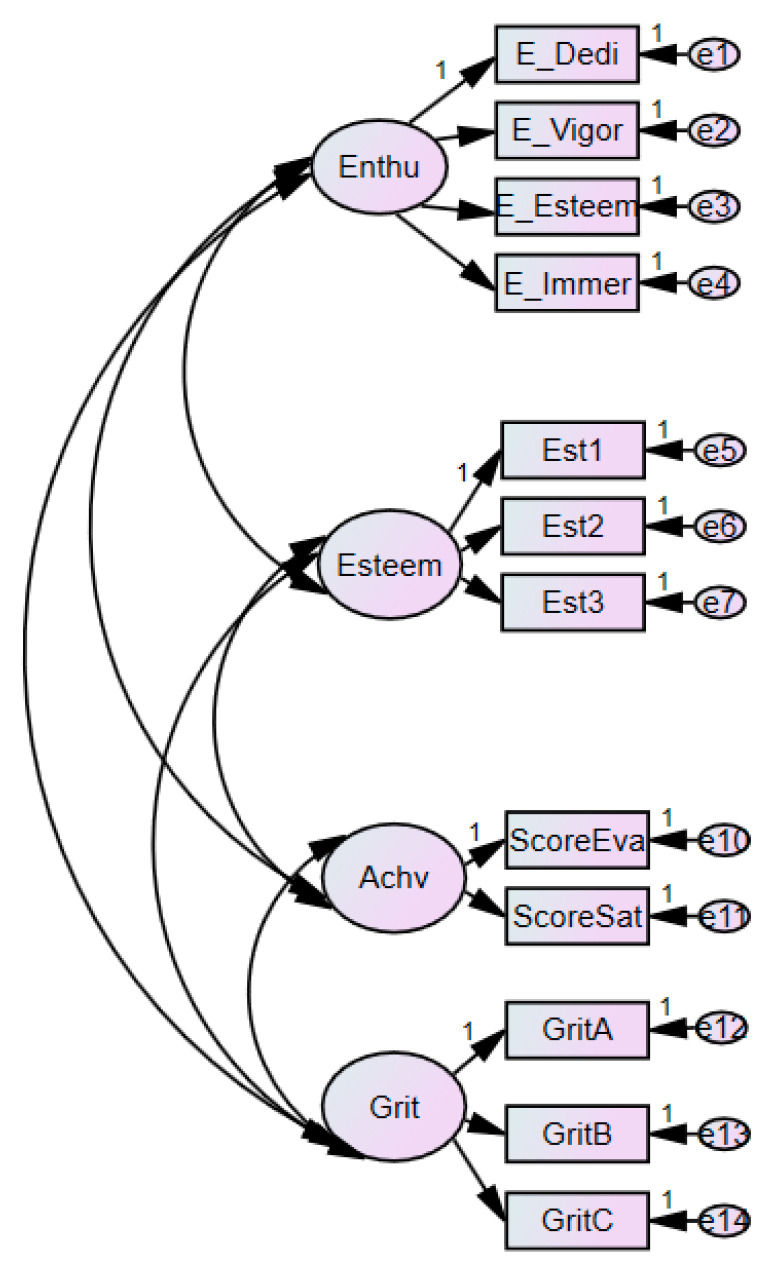
Confirmative factor analysis.

Confirmative factor analysis was conducted to verify utility of the measuring model in this study. In order to be simplistic and accurate, three sub-categories were set up on self-esteem and GRIT through data parceling, while setting three and two sub-categories for each of them, based on theoretical background. As shown in Figure 2, confirmative factor analysis was conducted on the research model. This research confirmed whether measuring variables and potential variables were well-organized, while examining if it was the right fit (Table 1). Using RMSEA (Root Mean Square Error of Approximation: 0.1 or below acceptable), CFI (Comparative Fit Index: 0.9 or above acceptable), and NFI (Normed Fit Index: 0.9 or above acceptable) that frequently used the goodness of fit as a criterion of evaluation for a model made for confirmative factor analysis [64,65], it was confirmed whether the measuring model was fit for the study.

### 2.4. Data Analysis and Research Procedures

This study was analyzed using SPSS21.0 (IBM, New York, NY, USA), AMOS22.0 (IBM, New York, NY, USA), and PROCESS macro program (Andrew F. Hayes, Alberta, Canada). Analysis is proceeded as follows.

First, confirmative factor analysis was conducted to confirm the level of appropriateness of measuring variables. Second, frequency analysis was conducted on independent, dependent, and mediator variables. Third, correlation analysis was conducted to identify the direction of relevant and relation among variables.

Fourth, the research model and alternative model were set up to choose a better model for the study, and the best model was chosen in the study. Fifth, the structural equation was set up to conduct covariance structural analysis, and it was intended to confirm the influence between variables in the study as well as the goodness of fit of the research model.

Sixth, mediator analysis was verified by using PROCESS macro. According to Lee, H.E. [66], PROCESS macro is an analytical method for verification of mediation effect and control effect without having to go through separate procedures by using regression analysis. PROCESS macro uses bootstrap to verify mediation effect, while setting up the basic number of samples as 2000 and proceeding the resulting value from the analysis in a single course. Therefore, it is more convenient to use without having to go through the analytic method by Baron and Kenny [67] or the Sobel test.

## 3. Results

Frequency analysis and correlation analysis were conducted as shown in Table 2 to confirm the relationship between variables. With this analysis, there was positive correlation between self-esteem, GRIT, academic enthusiasm, and academic achievement.

Next, each model was evaluated whether it was the right fit and compared by setting up how the full mediation model as an alternative model and the partial mediation model as a research model competed with each other. The comparison of this research model and alternative model was driven from Kelloway [68]. In his study for how it was required to compare the goodness of fit of a model in the partial mediation model and full mediation model to confirm whether the mediation model was the most appropriate model.

Partial mediation model has set up a hypothesis that vocational calling provided connection of influence on the career-performance in the medium of social capital. The index of fit of the partial mediation model is suggested in Table 3. The partial mediation model was significant with χ^2^ = 1074.864 (df = 45, *p* < 0.001), and index of fit was NFI = 0.902, CFI = 0.906, RMSEA = 0.094 representing proper index of fit in this study [64,65].

During this next analysis, the alternative model of full mediation model was established to compare it with a partial mediation model. To make the full mediation model to compare with the model fit of partial mediation model, the arrow in direct relationship between self-esteem and academic achievement was deleted.

Index of fit of the full mediation model was significant with χ^2^ = 1075.741 (df = 46, *p* < 0.001) as shown in the Table 3 and represented an acceptable level of goodness of fit with NFI = 0.902, CFI = 0.906, RMSEA = 0.094 [64,65]. When compared with the partial mediation model, the full mediation model turned out to represent the same level of index of fit.

**Figure 3:**
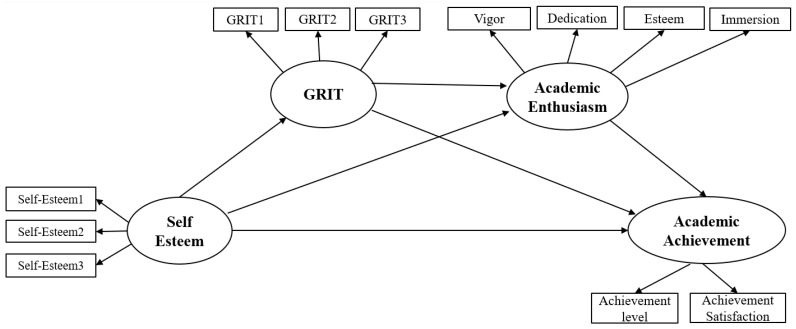
Partial mediation model.

**Figure 4:**
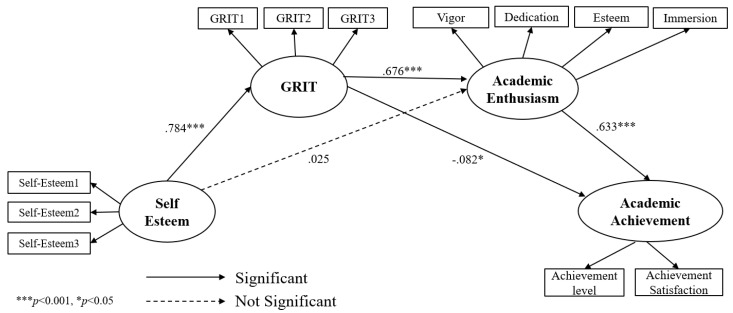
Full mediation model.

Next, a model comparison was conducted through the difference of values between the two models to compare the alternative model and research model. The result showed the difference of χ^2^ turned out to be 0.877 which was smaller than the threshold of 3.84 with the difference of 1 df. Therefore, it was not significant. When comparing values and degree of freedom of the two models, model verification did not reveal significant results. In this case, the model with higher degree of freedom tends to be selected [68]. In the comparison between the full mediation model and partial mediation model, the full mediation model with a higher degree of freedom was selected. In other words, the full mediation model was determined to be more appropriate than the partial mediation model through the comparison of goodness of fit and the difference of χ^2^. In Figure 3, it suggested the partial mediation model as a research model. In Figure 4, it suggested the full mediation model as an alternative model.

In order to analyze the relationship between major variables in this study, Standardized Path Estimates (β), Standard Error (S.E), *t*-value (C.R), and *p*-value were reviewed in the full mediation model. The results of analysis are shown as follows in Table 4 and Figure 4. The influence of self-esteem on GRIT turned out positive with β = 0.784 (*p* < 0.001). The influence of GRIT on academic enthusiasm was significant with β = 0.676 (*p* < 0.001). In addition, the influence of academic enthusiasm on academic achievement turned out to be significant with β = 0.633 (*p* < 0.001). On the other hand, the relationship between GRIT and academic achievement showed a negative relationship with β = −0.082 (*p* = 0.031), and the relationship between self-esteem and academic enthusiasm turned out to be of significance (*p* = 0.676).

Finally, the model number 6 in PROCESS macro [16] was applied to conduct the analysis. The result revealed an indirect effect between self-esteem as the independent variable, and GRIT and academic enthusiasm as parameters, and academic achievement as the dependent variable turned out to be 0.3388, as shown in Table 5. In addition, since the value in the 95% of confidence level did not include 0, the mediation effect turned out to be significant.

## 4. Discussion

This study aimed to identify the influence of self-esteem on academic achievement on 2590 first graders in middle school among data from the Korean Children and Youth Panel Survey 2018 and to verify the mediation effect of GRIT for mental care and academic enthusiasm in the influence of self-esteem on academic achievement.

According to the results of research, self-esteem among middle school students turned out to represent a statistically significantly positive relationship in academic achievement through GRIT and academic enthusiasm. In addition, with this study, it was confirmed that self-esteem ended up indirectly influencing academic achievement through GRIT and academic enthusiasm instead of directly influencing it. Discussion and conclusion are as follows, based on aforementioned results in the study.

First, the influence of self-esteem of middle school students on GRIT turned out to be statistically significant. Additionally, it was found that middle school students perceived to have high GRIT had statistically high academic enthusiasm, and academic enthusiasm had a significantly positive influence on academic achievement. These results showed a similar conclusion obtained by Duckworth et al. [19] which showed that self-esteem was related to academic achievement and also correlated with academic self-efficacy among first graders in middle school. In this study, it was found that the higher the level of self-esteem among middle school students, the more likely it was for them to positively evaluate themselves, which reinforced high levels of academic achievement. Therefore, there is a need to develop specific programs to improve self-esteem, GRIT, and academic enthusiasm as variables closely related to academic achievement of middle school students.

Second, GRIT and academic enthusiasm turned out to provide a connection in the influence of self-esteem on academic achievement. According to the research by Seo et al. [10] showing that the higher the self-esteem was, the more likely it was for students to improve learning motivation and achievement, it was not possible to know the mediator variable in the mid-process. In this study, the successive process for how self-esteem influenced GRIT, and GRIT influenced academic enthusiasm, was revealed. Therefore, continuous research is required along with the approach on various variables on self-esteem.

Third, the relationship between GRIT and academic achievement turned out to be negative in this study. In addition, there was a result that the relationship between self-esteem and academic enthusiasm was not significant. This explains how self-esteem and academic enthusiasm are not directly related, while self-esteem improves the level of GRIT, and GRIT enhances academic enthusiasm of middle school students. Seen in this perspective, there is a need to look for a new role for GRIT to be more actively involved, help students improve self-esteem through consultation for advancing to college, and realize GRIT at the same time so that career consulting instructors support middle school students to overcome environmental changes such as failure, adversity, and psychological pain and continue making an effort for their goal after experiencing failure on academic achievement. In addition, it is required to make an effort on institutional support such as administrative and financial support from the department of education and the office of education on classroom teachers, consulting instructors, or career consulting instructors in middle school.

As the result of the study on the relation between GRIT and academic achievement by Slick and Lee [68] targeting international students in Korea showed, GRIT had an negative correlation with giving up on their studies in the middle. Furthermore, a study of GRIT and academic achievement targeting Korean university students showed a significant positive correlation between GRIT and academic achievement in [16]. In line with previous studies [20], which showed a significant correlation between GRIT and academic performance, GRIT and academic performance also showed a strong positive correlation.

According to aforementioned results of the study, limitations of this study and several suggestions for follow-up research are as follows. First, this study aimed to comprehensively examine the influence on academic achievement according to correlation among elements by focusing on individual internal variables. However, since there are various external variables influencing academic achievement in the adolescent period such as family environments, friends, or schools, there is a need to explore such variables. Then, it might be difficult to generalize the questionnaires in the survey or results in this study as analysis was conducted by using panel data. Therefore, more meaningful analysis can be conducted if qualitative research is proceeded to identify self-esteem on academic achievement if considering parental support or the relationship between instructors and students later.

## Figures and Tables

**Table 1:** Goodness of fit indexes for the structural equation model.

χ^2^	df	CFI	NFI	RMSEA	*p*
1074.864	45	0.906	0.902	0.094	0.000

**Table 2:** Means, standard deviations, Cronbach’s α, and correlations of study variables.

Variables	Mean	Standard Deviation	Cronbach’s α	1	2	3	4
1. Self-esteem	3.485	0.503	0.866	-			
2. GRIT	3.159	0.439	0.712	0.524 *	-		
3. Academic enthusiasm	2.472	0.552	0.929	0.454 *	0.494 *	-	
4. Academic achievement	3.532	0.959	0.751	0.248 *	0.238 *	0.412 *	-

* *p* < 0.001.

**Table 3:** Goodness of fit indexes for partial mediation model and full mediation model.

Model	χ^2^	df	CFI	NFI	RMSEA	*p*
Partial Mediation Model	1074.864	45	0.906	0.902	0.094	0.000
Full Mediation Model	1075.741	46	0.906	0.902	0.094	0.000

**Table 4:** The path estimates and the effect decompositions of the research model.

Path	Standardized Path Estimates	Standard Error	C.R.	*p*
Self-esteem → GRIT	0.784	0.022	20.331	0.000
GRIT → Academic enthusiasm	0.676	0.110	9.284	0.000
Self-esteem → Academic enthusiasm	0.025	0.052	0.418	0.676
Academic enthusiasm → Academic achievement	0.633	0.078	13.801	0.000
GRIT → Academic achievement	−0.082	0.098	−2.153	0.031

**Table 5:** PROCESS macro analysis results.

Path	Indirect Effect	LLCI	ULCI
Self-esteem → GRIT → Academic enthusiasm → Academic achievement	0.3388 *	0.2847	0.3961

* *p* < 0.001.

## Data Availability

The Korea Children and Youth Panel Survey data are available in the NYPI Youth and Children Data Archive website (www.nypi.re.kr, accessed on 20 May 2021).
